# Effects of 12-Week Dietary Inflammatory Index-Based Dietary Education on Frailty Status in Frail Patients with Colorectal Cancer: A Randomized Controlled Trial

**DOI:** 10.3390/nu17132203

**Published:** 2025-07-01

**Authors:** Yuting Wang, Yuan Liu, Lan Cheng, Jianyun He, Xinxin Cheng, Xiaoxia Lin, Xinyi Miao, Zhenzhen Huang, Shufang Xia

**Affiliations:** Wuxi School of Medicine, Jiangnan University, Wuxi 214122, China; wangyuting@stu.jiangnan.edu.cn (Y.W.); 6212807025@stu.jiangnan.edu.cn (Y.L.); chenglan@stu.jiangnan.edu.cn (L.C.); hejianyun@stu.jiangnan.edu.cn (J.H.); chengxinxin@stu.jiangnan.edu.cn (X.C.); linxiaoxia@stu.jiangnan.edu.cn (X.L.); miaoxinyi@stu.jiangnan.edu.cn (X.M.); huangzhenzhen@stu.jiangnan.edu.cn (Z.H.)

**Keywords:** colorectal cancer, frailty, dietary inflammatory index, dietary education, inflammation

## Abstract

**Background**: Frailty is common in colorectal cancer (CRC) patients and is associated with poor prognosis and increased mortality. Anti-inflammatory dietary education is a promising and cost-effective strategy for frailty improvement. **Methods**: A prospective, assessor-blinded, two-arm randomized controlled trial was conducted to assess the effects of a 12-week dietary inflammatory index (DII)-based anti-inflammatory dietary education program on frailty in frail CRC patients. Participants in the intervention group received a DII-based anti-inflammatory dietary education, while the control group received a routine health education. Outcome measurements included the Fried frailty phenotype (FP), DII, plasma inflammatory biomarkers, body mass index (BMI), nutritional status, and quality of life (QoL), which were all assessed at baseline and post-intervention. **Results**: A total of 86.4% (57/66) of participants completed the follow-up. No statistically significant baseline differences were observed between groups. After the intervention, the intervention group showed significant improvements in DII (*p* = 0.029), BMI (*p* = 0.012), mini nutritional assessment (MNA) scores (*p* = 0.027), and QoL (*p* = 0.014) compared with the control group. Within-group comparisons revealed significant decreases in frailty status (*p* = 0.031), DII (*p* = 0.008), and interleukin (IL)-6 (*p* = 0.003), and significant increases in IL-10 (*p* = 0.021), MNA scores (*p* = 0.010), and QoL (*p* < 0.001) in the intervention group, with no significant changes in the control group. **Conclusions**: DII-based anti-inflammatory dietary education can improve the frailty, nutritional status, and QoL of frail CRC patients by modulating systemic inflammation. Given its acceptability and utility, this strategy may be incorporated into routine cancer health education.

## 1. Introduction

Colorectal cancer (CRC) has become the third most common cancer and the second most deadly cancer among all malignancies worldwide [1]. It mainly occurs in the elderly, with peak incidence around the age of 80 [2]. The disease itself and the side effects of treatments, as well as the decline in physiological reserves with age, lead to frailty in CRC patients. Frailty is a complex syndrome involving multiple factors, characterized by reduced strength, endurance, and physiologic function, which increases an individual’s susceptibility to dependence and/or death [3,4]. Epidemiological data have shown that the prevalence of frailty is steadily increasing among CRC patients [5], reaching approximately 12% to 56% [6,7]. Although frailty increased the risk of adverse health outcomes in cancer patients, including dementia, disability, hospitalization, and mortality [8,9], it remains a dynamic and modifiable condition [10,11], suggesting that intervention can improve health outcomes for CRC patients.

Unfortunately, research to improve individual frailty is still in its infancy, especially for cancer patients. Risk factors for frailty are diverse and include sociodemographic factors, chronic diseases, psychological factors (depression and cognitive impairment), and lifestyle factors (malnutrition and physical inactivity) [12,13]. Dietary intervention is a kind of lifestyle modification and may result in improvements in frailty status. Several nutritional interventions, such as dietary supplements involvement and meal support, have been applied and apparently improve frailty status [14]. However, the disadvantage is that it is obviously costly, which may limit its long-term sustainability [15]. Instead, interventions including dietary education, such as promoting healthy dietary patterns and encouraging dietary behavior change, have reported positive outcomes [16,17]. Dietary interventions such as dietary plans, self-guided educational materials, individualized nutrition counseling, and group dietary courses, have effectively improved frailty status among older adults [18]. Another study reported that older adults with frailty or pre-frailty who received personalized dietary counseling for three months had reduced frailty scores, increased grip strength, and faster gait speed after shifting to the recommended dietary pattern [19]. We found no studies that targeted frail CRC patients to explore the effectiveness of dietary education on frailty.

Although the mechanism by which diet affects frailty in cancer patients is not yet clear, existing research suggests that inflammation may be involved. Nutritional status has been found to regulate the immune system by affecting cellular metabolism, which alters cytokine production, leading to inflammation and consequently frailty [20]. Different diets may present anti-inflammatory or pro-inflammatory effects. For example, diets rich in fruits and vegetables exert anti-inflammatory properties, whereas high-fat diets rich in saturated and trans fats are associated with pro-inflammatory effects. [21]. The dietary inflammatory index (DII) was a reliable assessment tool to evaluate an individual’s overall dietary inflammatory potential [22]. Our previous study revealed a positive association between DII scores and the risk of frailty among patients with CRC, potentially mediated by interleukin (IL)-6 and IL-10 [23]. For CRC patients, due to the lack of post-diagnosis dietary guidance, they often contribute to unhealthy dietary habits, characterized by excessive intake of red and processed meats and insufficient consumption of fruits and vegetables, leading to increased dietary pro-inflammatory potential. A longitudinal study has shown that these unhealthy dietary habits were correlated with decreased quality of life (QoL) and increased symptom burden [24]. Thus, DII-based dietary interventions might have the potential to ameliorate frailty in CRC patients by inhibiting systemic inflammation. Considering that dietary education plays a key role in shaping patients’ eating behaviors and improving their nutritional status [25], it is more cost-effective and more likely to be widely implemented.

The objective of this study was to examine the effects of a 12-week DII-based anti-inflammatory dietary education program on the improvement of frailty in CRC patients undergoing chemotherapy. We hypothesized that after the intervention, participants in the intervention group would show improvement in frailty, a lower DII score, reduced systemic inflammation, and enhanced QoL.

## 2. Materials and Methods

### 2.1. Design Overview

This single-center, assessor-blinded, two-arm randomized controlled trial (RCT) was approved by the Medical Ethics Committee of the Affiliated Hospital of Jiangnan University (Approval No. LS2024543) and prospectively registered with the Chinese Clinical Trial Registry (ChiCTR2500097644). The study protocol was conducted in accordance with the ethical principles of the Declaration of Helsinki. The trial was reported in compliance with the Consolidated Standards of Reporting Trials (CONSORT) statement guidelines.

### 2.2. Participants

From December 2024 to January 2025, CRC patients were recruited from the Department of Oncology in the Affiliated Hospital of Jiangnan University. The inclusion criteria included: (1) had a diagnosis of stage I-III colorectal cancer; (2) completion of curative resection with planned adjuvant chemotherapy (≥ 4 cycles); (3) frailty phenotype defined by the Fried frailty criteria (score ≥ 3); (4) age ≥ 18 years; (5) normal cognitive function and communication abilities; (6) possessed a smartphone for message reception and communication; (7) having at least one caregiver; (8) voluntary participation in the study. The exclusion criteria were: (1) colorectal cancer with recurrence or distant metastasis; (2) history of other malignancies; (3) history of frailty targeted interventions within 3 months prior to enrollment; (4) recent exposure to antibiotics or immunomodulatory medications; (5) concurrent enrollment in any Good Clinical Practice (GCP)-regulated clinical trial; (6) presence of severe somatic or organic brain comorbidities; (7) active or clinically significant infections.

### 2.3. Sample Size Calculation

The sample size estimation followed a standard formula:

$$n=(Z_{\alpha }+Z_{\beta }{)}^{2}\times 2\sigma^{2}/\delta^{2}$$

The mean difference (δ) in Fried frailty scores between the intervention and control groups was 0.7, and a pooled standard deviation (σ) was 0.82. Assuming a two-tailed significance level (α) of 0.05 and statistical power (1 − β) of 0.8, the calculated sample size was 22 participants per group. The final recruitment target was set at 28 participants per group, taking into account a 20% attrition rate.

### 2.4. Randomization and Masking

An independent researcher who did not participate in the study used the Research Randomizer website (https://www.randomizer.org/, accessed on 18 December 2024) to generate a randomized allocation sequence, which was then sealed in serially numbered opaque envelopes. Another independent researcher assessed baseline data on eligible participants who signed informed consent forms, then subsequently opened the envelopes for random assignment in the order of enrollment, ensuring equal allocation to the intervention and control groups. Group allocation remained concealed from outcome assessors and data analysts throughout the study. After randomization, participants received DII-based dietary education during hospitalization and were invited to a WeChat group for nutritional support. However, due to the nature of dietary education, participants and interventionists could not be blinded. To reduce the risk of bias, several measures were implemented: (1) upon admission, we first assessed frailty status and allocated the frail patients to wards with non-frail patients, ensuring that there was at most one frail participant per ward; (2) we provided specialized training for outcome assessors to emphasize neutrality during the assessment process; (3) assessments were conducted in dedicated rooms to minimize intervention-related interference; (4) anonymized sample IDs were used to mask the group allocations when the laboratory technicians tested inflammatory biomarkers.

### 2.5. Intervention

At baseline and following the intervention, patients received a pre-admission 24-h dietary recall survey, regardless of group allocation. In order for participants to understand and gain knowledge of the DII and apply it to home dietary practices to improve dietary behaviors, patients in the intervention group were provided with a DII-based dietary education session during each hospitalization, as well as 12 weeks of nutritional support. Trained interventionists provided in-person educational sessions to participants and their caregivers in a dedicated room to improve patients’ anti-inflammatory dietary potential by encouraging participants to adhere to a dietary pattern that emphasized limiting pro-inflammatory foods and enhancing anti-inflammatory dietary components [26]. Specifically, dietary recommendations included increasing the intake of fruits, vegetables, legumes, nuts, seeds, whole grains, and healthy fats such as olive oil, along with moderate amounts of eggs and low-fat dairy products, seafood, and lean poultry for protein sources. Participants were advised to limit their intake of red meat, processed meat (such as hot dogs, luncheon meats, and bacon), sugary foods and beverages (including sugar-sweetened soft drinks), refined carbohydrates, fried foods, margarine, shortening, and other products containing partially hydrogenated oils. Personalized feedback was provided to participants after dietary data was analyzed. Feedback included comparing the difference between the patients’ actual dietary intake and the recommended intake outlined in the Chinese Dietary Guidelines for Chinese residents (2022) and providing personalized dietary recommendations based on participants’ dietary patterns, as summarized in Supplementary Table S3. Finally, participants and their caregivers were encouraged and guided to keep written dietary records or photographs of meals. Upon completion of the initial dietary education session, participants received a DII-based dietary guidance brochure to facilitate their daily dietary decision-making. The brochure covered the following topics: (1) symptoms and adverse consequences of frailty; (2) associations between DII and frailty; (3) visual charts of pro-inflammatory and anti-inflammatory foods; (4) recommended dietary intakes based on the Dietary Guidelines for Chinese Residents; and (5) methods of dietary recording. During the home-based intervention period, researchers provided online nutritional counseling support through WeChat or telephone to address their dietary queries, reminded them to keep dietary records, and encouraged them to share experiences in online groups to enhance peer communication and active participation. Subsequently, personalized feedback was provided to each participant based on the dietary records, offering praise for improved eating behaviors and solutions to existing unhealthy dietary behaviors. Additionally, caregivers were instructed to supervise and remind participants to follow dietary recommendations.

Participants in the control group were offered routine disease-related health education, including four in-person educational sessions in the hospital and 12 weeks of nutritional support via WeChat or telephone after discharge. The in-person education included knowledge of CRC disease and treatment such as current epidemiological characteristics, risk factors and treatments, management of common chemotherapy-related adverse effects, care and maintenance of infusion ports, and recommendations for regular post-treatment follow-up assessments. Interventionists did not offer unsolicited dietary guidance and only made cursory recommendations, such as increasing or decreasing intakes of certain food groups, in response to patients’ individual dietary findings when they asked for dietary recommendations, but did not involve any education on anti-inflammatory or pro-inflammatory diets. For online support, researchers assisted participants by providing reminders for treatment appointments and addressing patient counseling.

To minimize the dropout rate, we recruited CRC patients who were expected to receive at least four cycles of adjuvant chemotherapy, ensuring that they were still in the inpatient phase of treatment requiring chemotherapy after the 12-week intervention to facilitate questionnaire surveys and blood sample collection. Additionally, two days prior to each admission for chemotherapy, the researchers called patients to remind them of their upcoming treatment appointments to ensure that they would not miss them. To encourage patients to attend follow-up visits, patients who completed each intervention received a small reward, such as a water cup, massage hammers (percussion massagers), canvas bags, or mini calendars.

### 2.6. Sociodemographic and Clinical Information

At baseline, sociodemographic data and disease characteristics of the participants were collected. Considering that key lifestyle behaviors, including physical activity, smoking, and alcohol consumption are critical determinants of frailty status [27,28], these factors were also assessed. Physical activity levels were evaluated using the International Physical Activity Questionnaire Short Form (IPAQ-SF), which categorizes participants’ physical activity into three levels based on metabolic equivalents (METs): low (<600 MET-min/week), moderate (600 to 1500 MET-min/week), and high physical activity (>1500 MET-min/week) [29]. Data collection was conducted by trained nutritionists, dietitians, phlebotomists, and research assistants.

### 2.7. Primary Outcome

Frailty status was assessed with the Fried frailty phenotype (FP) scale [30]. Its Chinese version has been validated with a Cronbach’s α coefficient of 0.93 [31]. This tool defines five criteria: unintentional weight loss, self-reported exhaustion, weakness (grip strength), slowness (walking speed), and low physical activity level. A detailed description of the FP scale is provided in Table S1. Patients with a score ≥ 3 were classified as frail and selected as study candidates. They were reassessed with the Fried frailty criteria upon completion of the 12-week intervention.

### 2.8. Secondary Outcomes

#### 2.8.1. Dietary Intake Assessment and DII Calculation

Dietary intake was assessed at baseline and upon completion of the intervention using a 3-day 24-h dietary recall that included food types, quantities, and preparation methods on one weekend and two workdays [32]. To enhance the accuracy of dietary surveys, food models and food atlases were used to assist participants or caregivers in estimating food intake at the time of patient hospitalization. Dietary intake was collected for the past 24 h after admission. The remaining two dietary recalls were collected via WeChat following hospital discharge and the resolution of chemotherapy-related gastrointestinal symptoms. Participants or their caregivers used kitchen scales to weigh food, oils, and condiments, and reported intake through photographs or video calls. Nutrition Calculator v2.7.8.8 (Chinese Center for Disease Control and Prevention, Beijing, China) was used to calculate daily nutrient intake.

The detailed DII score calculation process was reported in our previous publication [23], in which 25 dietary parameters were used, including energy, protein, fat, carbohydrates, dietary fiber, cholesterol, vitamin A, vitamin D, vitamin E, vitamin B1, vitamin B2, vitamin B6, vitamin C, folate, niacin, magnesium, iron, zinc, selenium, β-carotene, SFA, monounsaturated fatty acids (MUFA), polyunsaturated fatty acids (PUFA), ω-3, and ω-6 fatty acids. The DII score calculated with 25 to 30 parameters theoretically ranges from −5.5 to 5.5 [33]. The DII quantifies the inflammatory potential of the diet, whereby higher scores reflect more pro-inflammatory potential, and lower scores indicate more anti-inflammatory potential.

#### 2.8.2. Plasma Inflammatory Biomarkers

Fasting blood samples were collected from 56 participants at baseline and before the start of chemotherapy at week 12. The samples were centrifuged to prepare plasma, which was subsequently stored at −80 °C. According to our previously findings [23], both IL-6 and IL-10 were mediators in the association between DII and frailty in CRC patients. These two interleukins were measured using enzyme-linked immunosorbent assay (ELISA) kits (Meimian, Yancheng, China).

#### 2.8.3. Nutritional Status Assessment

The nutritional status of the participants was assessed using the Mini Nutritional Assessment (MNA) scale. The scale can be grouped into four rubrics to obtain sub-scores: anthropometric assessment, general assessment, short dietary assessment, and subjective assessment. The MNA has a maximum score of 30, with a score ≥24 suggesting good nutritional status, a score between 17 and 23.5 indicating a risk of malnutrition, and a score ≤16.5 indicating malnutrition [34].

#### 2.8.4. Quality of Life

The Functional Assessment of Cancer Therapy-Colorectal (FACT-C) scale was used to assess the QoL [35]. It consists of seven items each for physical well-being, social/family well-being, emotional well-being, and 12 additional items, ranging from 0 to 136. A higher total score indicates better QoL for the patient. The Chinese version has been validated with Cronbach’s α coefficients higher than 0.80 for all domains and the total module, except for the additional concerns domain, which had a coefficient of 0.56 [36].

### 2.9. Statistical Analysis

Statistical analyses were performed using SPSS 27.0 (IBM, Chicago, IL, USA). Continuous variables were tested for normality using the Shapiro–Wilk test and expressed as mean ± standard deviation (SD) or median (25%, 75% percentiles), followed by an independent-sample *t*-test and a Mann–Whitney U test for between-group comparisons, respectively. Categorical variables were presented as frequencies and percentages (*n*, %) and analyzed by a Chi-squared test, continuity-corrected Chi-squared test, or Fisher’s exact test. For within-group comparisons, paired sample *t*-tests and Wilcoxon signed-rank tests were used to analyze the differences before and after the intervention, respectively. A two-sided *p*-value < 0.05 was considered statistically significant.

## 3. Results

### 3.1. Overview

**Figure 1:**
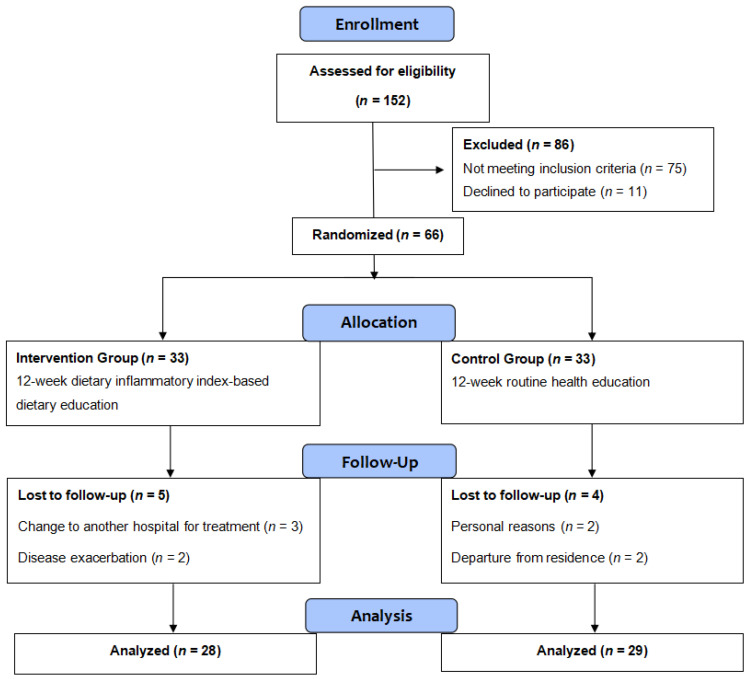
CONSORT flow diagram of participant recruitment during the trial.

One hundred and fifty-two CRC patients were screened, of whom 75 (49.4%) did not meet the eligibility criteria, 11 (7.2%) refused participation, and the remaining 66 frail CRC patients were randomly assigned to either the intervention group or control group, with 33 participants in each group. During the period of the study, three participants in the intervention group changed hospitals for treatment and two participants withdrew due to exacerbation. In the control group, two participants left their place of residence and two withdrew for personal reasons. Thus, after 12 weeks of intervention, data were collected from 57 participants (28 in the intervention group and 29 in the control group). One participant in the control group refused to provide blood samples, so 28 participants in each group underwent measurement of plasma inflammatory markers. The flow diagram of the study is shown in Figure 1.

### 3.2. Baseline Characteristics

As shown in Table 1, Table 2 and Table 3, the mean age of participants was 68.75 ± 6.13 years, with a mean BMI of 21.37 ± 2.85 kg/m^2^. The majority of patients were male (57.9%) and diagnosed with stage III tumors (54.4%). The vast majority of patients reported moderate physical activity (64.9%). There were no statistically significant differences in baseline characteristics between the two groups (*p* > 0.05).

### 3.3. Effects on Frailty Status

**Figure 2:**
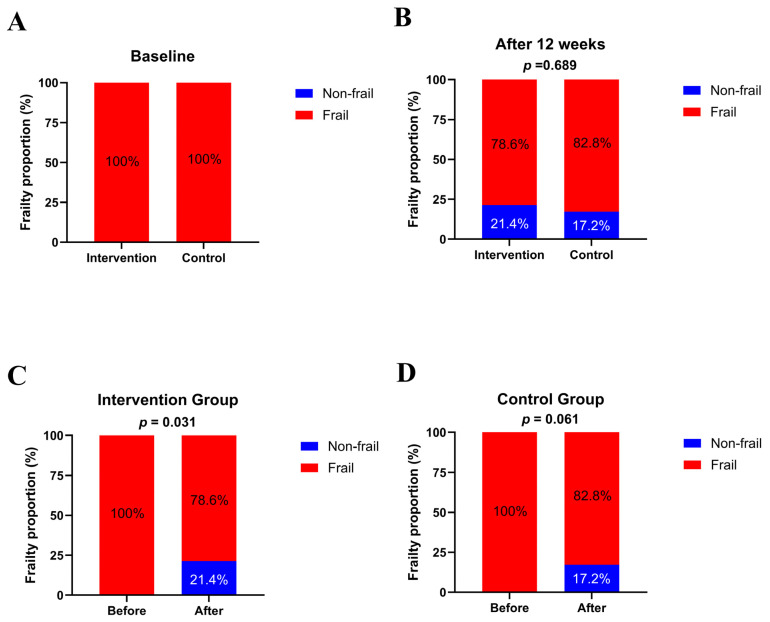
Effects of the 12-week dietary inflammatory index-based dietary education on frailty status in frail patients with colorectal cancer and receiving chemotherapy. (**A**,**B**): Frailty proportion of intervention and control groups at baseline and after 12 weeks. Data are presented as percentages. Chi-squared test was used. (**C**,**D**): Frailty proportion of intervention and control groups from baseline to the end of follow-up. Chi-squared test with continuity correction was used.

At baseline, both groups of CRC patients were frail (Figure 2A). After the 12-week intervention, there was no statistically significant difference in the prevalence of frailty between the intervention and control groups (*p* = 0.689; Figure 2B). The proportion of frail patients in the intervention group decreased significantly compared with the baseline (*p* = 0.031; Figure 2C), whereas the prevalence of frailty in the control group showed a decreasing trend compared with the baseline, which was not statistically significant (*p* = 0.061; Figure 2D).

The frailty scores of the control and intervention groups are shown in Table S2. No statistically significant differences were observed in FP scores between the two groups, both pre- and post-intervention (*p* > 0.05). Furthermore, after 12 weeks of intervention, there were no significant changes in FP scores compared to baseline in either the intervention or control group (*p* > 0.05).

### 3.4. Effects on Dietary Inflammatory Potential

**Figure 3:**
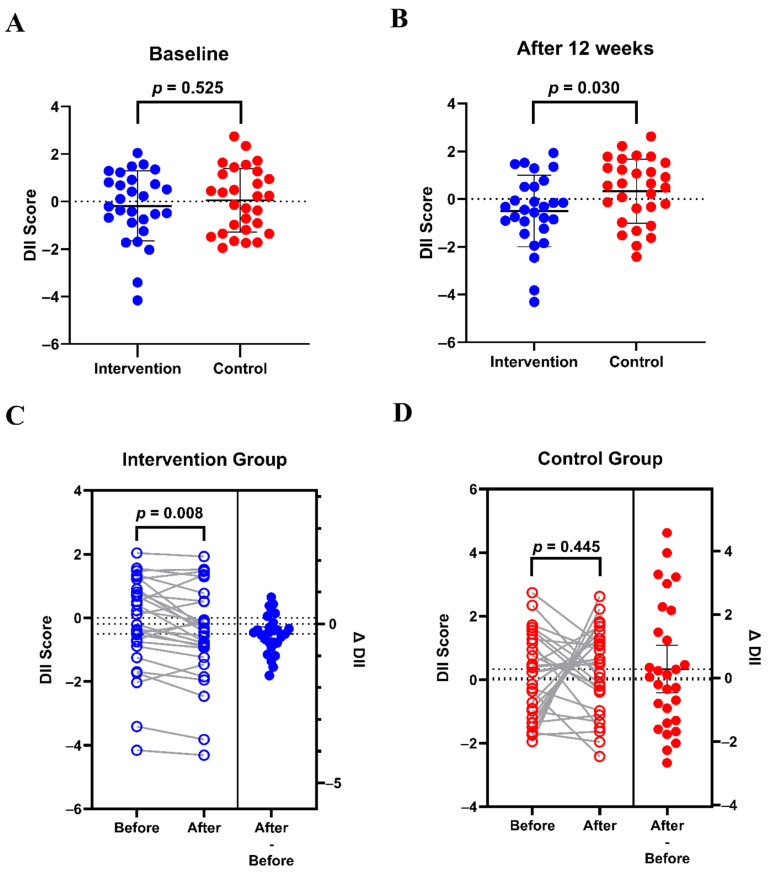
Effects of the 12-week dietary inflammatory index-based dietary education on DII score in frail patients with colorectal cancer and receiving chemotherapy. (**A**) DII scores at baseline; (**B**) DII scores after 12 weeks. Data are presented as mean ± SD. Independent samples *t*-test was used. (**C**) DII scores of the intervention group from baseline to the end of follow-up; (**D**) DII scores of the control group from baseline to the end of follow-up. Paired *t*-test was used. DII, dietary inflammatory index. The change (∆) was defined as the value after the intervention minus the value at baseline for the same individual. The blue circles represent the intervention group, and the red circles represent the control group.

At baseline, the DII scores between the control and intervention groups of frail CRC patients were not significantly different (*p* = 0.525; Figure 3A). After the 12-week intervention, the intervention group showed significantly lower DII scores compared to the control group (–0.50 ± 1.49 vs. 0.33 ± 1.33; *p* = 0.030; Figure 3B). DII scores in the intervention group were significantly decreased compared with the baseline (∆ DII = –0.32 ± 0.59; *p* = 0.008; Figure 3C), whereas the control group exhibited a non-significant increase in DII scores compared with the baseline (∆ DII = 0.28 ± 1.96; *p* = 0.445; Figure 3D).

### 3.5. Effects on Plasma Inflammatory Biomarkers, BMI, Nutritional Status, and Quality of Life

As shown in Table 4, Table 5 and Table 6, at baseline, there were no significant differences in plasma IL-6 and IL-10 levels, BMI, MNA, and FACT-C scores between the intervention and control groups (*p* > 0.05). After the 12-week intervention, although there were no significant differences in IL-6 and IL-10 levels between the two groups (*p* > 0.05), the intervention group exhibited a significantly greater reduction in IL-6 levels and a significantly higher increase in IL-10 levels compared to the control group (*p* < 0.05). Additionally, the BMI, MNA, and FACT-C scores, as well as the change values of BMI and MNA scores, were all significantly higher in the intervention group than in the control group (*p* < 0.05). Compared with baseline data, there were no significant changes in IL-6, IL-10, BMI, MNA, and FACT-C scores in the control group after the intervention (*p* > 0.05), but there was a significant decrease in IL-6 levels and a significant increase in IL-10, BMI, MNA, and FACT-C scores in the intervention group (*p* < 0.05).

## 4. Discussion

This 12-week randomized controlled trial provided evidence that a DII-based anti-inflammatory dietary education intervention significantly improved frailty, nutritional status, and QoL in frail CRC patients receiving adjuvant chemotherapy, suggesting that this strategy might be beneficial in enhancing physiologic reserves and tolerance to disease treatment and contributing to improved health outcomes in cancer patients.

Although studies have emphasized the crucial role of nutritional interventions in reversing frailty and preventing adverse outcomes when frailty occurs [37], existing studies have mainly focused on the elderly. For example, a multicenter study across five European countries found that a one year Mediterranean dietary intervention significantly improved frailty and reduced inflammation in older people [38]. Another study from South Korea illustrated that an elderly-friendly dietary intervention lasting five months led to modest improvements in frailty, with significant differences emerging over a longer follow-up period [39]. To date, few studies have examined the effects of nutritional interventions on frailty in cancer patients. In the present study, although the proportion of frail patients at 12 weeks showed no significant difference between groups, DII-based anti-inflammatory dietary education was effective in improving frailty in CRC patients compared with baseline data. The reason for this limited difference observed could be associated with the intervention cycle. Many studies of dietary interventions on frailty in older adults have had at least one year of follow-up, which was much longer than our 12-week follow-up period. In addition, to ensure that data could be collected from patients after the intervention, we recruited patients during the chemotherapy phase and ensured that chemotherapy had not yet been completed at the end of the intervention, which meant that throughout the intervention phase each patient still had to suffer from the negative effects of chemotherapy, such as reduced physiological reserve and increased risk of frailty [40], which could result in the beneficial effects of the dietary intervention being offset by the chemotherapy. In the future, we should extend the follow-up time to one or even two years after the intervention to assess the role of adherence to a long-term anti-inflammatory diet on frailty of CRC survivors.

In our study, the 12-week DII-based dietary education demonstrated positive effects in enhancing dietary anti-inflammatory potential, suggesting that the frail CRC patients in this group were likely to consume less pro-inflammatory components or more anti-inflammatory components, which was consistent with our previous research that anti-inflammatory dietary education also significantly enhanced dietary anti-inflammatory potential in depressed patients with breast cancer [41]. Studies have shown that cancer survivors generally have a strong desire for dietary guidance and require personalized dietary strategies to manage symptoms, while healthcare professionals provide limited or difficult to implement information on healthy diets after disease treatment [42]. After assessing the overall dietary inflammatory potential of the participants and comparing their dietary data with the recommended intake of the Chinese Dietary Guidelines for Chinese Residents, we took into account the dietary habits of the patients, local food characteristics and seasonal features, and provided patients with information about a variety of anti-inflammatory ingredients (e.g., colorful vegetables and fruits) that should be consumed more often. We also gave patients guidelines on pro-inflammatory ingredients (e.g., processed meats, excessive red meat) that should be avoided or minimized and formulated a dietary improvement plan to guide participants on how to select foods to help them address their dietary challenges in a timely manner. Additionally, in this study, caregivers were also educated to enhance their role in fostering a supportive family dietary environment that promotes adherence to the diet [43].

Dietary intervention, such as personalized nutritional counseling and support, is vital for enhancing nutritional status and reducing the risk of frailty in cancer patients [44], possibly due to healthy dietary patterns that provide higher densities of micronutrients, which could positively impact muscle mass and function [45]. Our findings showed that DII-based dietary education improved nutritional status, evidenced by higher BMI and MNA scores. DII was calculated based on dietary nutrients, including macronutrients, vitamins, minerals, and fatty acids, most of which have been demonstrated to affect muscle protein synthesis and thereby influence frailty. For example, ω-3 fatty acids have been demonstrated to reduce inflammation-mediated cellular stress to create a favorable cellular environment for enhanced muscle protein synthesis [46]. Indeed, as critical components of frailty diagnosis, grip strength, quadriceps muscle strength, walking speed, and walking endurance could be improved by a 6-week ω-3 PUFA supplementation in elderly individuals [47]. Vitamin D can also optimize energy metabolism pathways to supply adequate energy and substrates required for muscle protein synthesis [48]. Additionally, amino acids stimulated muscle protein synthesis and increased the cross-sectional area of muscle fibers, consequently reducing the risk of frailty [49]. The combined supplementation of leucine and vitamin D showed beneficial effects on muscle strength and physical function, including improved grip strength and gait speed in older adults [50]. In frail CRC patients, DII-based dietary education could exert cumulative effects of different food combinations to improve nutritional status, thereby potentially alleviating frailty.

Inflammation was an underlying pathophysiologic process linking diet and frailty [51]. Nutrition has been shown to influence pro-inflammatory molecular signatures [52] and anti-inflammatory dietary interventions have been shown to decrease IL-6 levels and increased IL-10 in adults [53]. Following a two-week intervention with a traditional Korean diet rich in anti-inflammatory components, obese women exhibited lower DII scores and higher IL-10 levels than the control diet group [54]. The role of inflammation in frailty has also been substantiated. Studies suggested that elevated concentrations of IL-6 predicted declines in muscle strength and increased risks of physical disability, both of which were critical components of frailty syndrome [55]. Meanwhile, frail patients exhibited a pro-inflammatory phenotype characterized by significantly higher serum IL-6 and IL-6/IL-10 ratio than non-frail individuals [56]. In this study, CRC patients experienced a decrease in plasma IL-6 levels and an increase in IL-10 levels after receiving DII-based dietary education. This is consistent with the results of our previous cross-sectional study that plasma IL-6 and IL-10 levels were the mediators linking DII and frailty in CRC patients [23]. However, patients in the control group had a tendency to increase IL-6 and decrease IL-10, possibly due to the ongoing chemotherapy-induced inflammation. Therefore, DII-based dietary education balances the intakes of foods with pro-inflammatory and anti-inflammatory components, facilitates a decrease in the overall dietary inflammatory potential, reduces systemic inflammation, and thereby improves frailty.

Notably, the benefits of dietary education on frailty may extend beyond biological mechanisms to include behavioral and psychological pathways. Evidence showed that such interventions could improve nutritional knowledge, increase vegetable intake and dietary diversity, and enhance self-efficacy [57]. Given that frailty is a complex, multidimensional condition involving physical, cognitive, psychological, and social dimensions, it is important to consider whether dietary intervention alone is sufficient. Recent evidence suggests that multi-component interventions might offer enhanced benefits. For instance, a cluster-randomized controlled trial conducted across 13 community centers in Taiwan reported that a 3-month intervention combining group-based nutrition education with physical exercise led to greater improvements in frailty status and working memory compared to exercise alone [58]. Moreover, a 6-month multicenter RCT in Spain, which integrated dietary guidance with physical activity, cognitive training, psychological support, and social engagement significantly improved physical function, sleep quality, and nutritional status in community-dwelling older adults [16]. These findings highlight the potential value of combining dietary education with other targeted strategies to address the multifaceted nature of frailty and provide a compelling direction for future research on sustainable intervention models.

Better QoL was associated with prolonged survival in cancer patients and served as a robust predictor of patient-reported outcomes related to overall and progression-free survival [59], and it could be improved by nutritional intervention. It has been reported that DII-guided nutritional intervention attenuated both nutritional status and QoL in lung cancer patients receiving chemotherapy [60]. A systematic review also indicated that most dietary interventions tended to enhance overall QoL and its subdomains among cancer survivors [61]. In accordance with these findings, we also found that CRC patients who received dietary education showed improved QoL. However, it is noteworthy that a dietary intervention study among breast cancer survivors found improvements in their QoL at a 6-month follow-up, but these benefits were not sustained at a 12-month follow-up [62]. This might be due to the fact that the first 6 months of the dietary intervention consisted of an intensive combination of food preparation assistance, informational seminars, motivational telephone counseling, and monthly newsletters, after which the intensity and frequency of the intervention decreased significantly. Although the short-term effectiveness of dietary education was demonstrated in this study, long-term adherence to dietary recommendations remains uncertain. Factors such as age, educational level, economic status, social support, health beliefs, and chemotherapy-related side effects may all influence adherence over time [63,64,65,66,67]. These factors are critical for ensuring the applicability and sustained effects of dietary interventions. These considerations also suggest that when implementing nutritional interventions for patients, extended follow-up is essential to focus on the long-term effects on various health outcomes.

Several limitations of this study should be acknowledged. First, the questionnaire data relied on subjective self-reports, which are susceptible to participant and assessor bias. Second, although food modeling and atlas were employed to facilitate dietary recall of participants, the possibility of recall bias cannot be excluded. Third, given that DII-based dietary education requires consistent adherence over time, the 12-week intervention and lack of extended follow-up in this study constrain any evaluation of its sustained effects. Fourth, owing to the characteristics of non-pharmacological intervention, it was not practical to blind participants and interventionists, so only the assessors were blinded. Fifth, this study focused solely on dietary education, without integrating other components such as exercise or psychological support, which may limit its ability to address the multifactorial nature of frailty. Finally, this study was conducted in only one center and the participants were recruited through convenience sampling, which may have limited the representativeness of the sample. In addition, the relatively small sample size of this study necessitates caution in generalizing these findings to a broader population or other clinical settings. Therefore, more rigorous study designs, the incorporation of multi-component interventions, more accurate and objective measurement tools, larger sample sizes, multicenter designs, and longer-term follow-up with dynamic data should be adopted in future studies to assess the sustainability of intervention effects.

## 5. Conclusions

This study demonstrated that DII-based anti-inflammatory dietary education could effectively improve frailty status and increase dietary anti-inflammatory potential among frail CRC patients. Additionally, it also has beneficial effects on inflammatory biomarkers, nutritional status, and overall QoL, highlighting its promising potential for clinical application in the management of frailty among CRC patients.

## Figures and Tables

**Table 1:** Comparison of baseline demographic characteristics between the intervention and control groups.

Variables	Overall (*n* = 57)	Intervention (*n* = 28)	Control (*n* = 29)	t/χ^2^/Z	*p*
Age (year) ^a^	68.75 ± 6.13	70.14 ± 6.30	67.41 ± 5.76	1.708	0.093
BMI (kg/m^2^) ^a^	21.37 ± 2.85	21.67 ± 2.72	21.08 ± 2.99	0.788	0.434
Sex ^b^					
Male	33 (57.9)	16 (57.1)	17 (58.6)	0.013	0.910
Female	24 (42.1)	12 (42.9)	12 (41.4)		
Marital status ^c^					
Married	47 (82.5)	25 (89.3)	22 (75.9)	0.968	0.325
Widowed/divorced/single	10 (17.5)	3 (10.7)	7 (24.1)		
Education level ^d^					
Primary school or lower	23 (40.3)	12 (42.9)	11 (38.0)	1.298	0.912
Middle school	22 (38.6)	10 (35.7)	12 (41.4)		
High/secondary school	11 (19.3)	6 (21.4)	5 (17.2)		
Junior college or higher	1 (1.8)	0 (0.0)	1 (3.4)		
Employment ^d^					
Employed	2 (3.5)	1 (3.6)	1 (3.4)	2.309	0.355
Unemployed	8 (14.0)	2 (7.1)	6 (20.7)		
Retired	47 (82.5)	25 (89.3)	22 (75.9)		
Residence ^d^					
Rural areas	15 (26.3)	9 (32.1)	6 (20.7)	2.222	0.373
Towns	6 (10.5)	4 (14.3)	2 (6.9)		
Urban areas	36 (63.2)	15 (53.6)	21 (72.4)		
Family monthly income ^d^					
<2000 CNY	24 (42.1)	11 (39.3)	13 (44.8)	3.562	0.154
2000~5000 CNY	27 (47.4)	16 (57.1)	11 (38.0)		
>5000 CNY	6 (10.5)	1 (3.6)	5 (17.2)		

Data are shown as *n* (%) or mean ± SD. ^a^ Independent samples *t*-test. ^b^ Chi-squared test. ^c^ Chi-squared test with continuity correction. ^d^ Fisher’s exact test. BMI, body mass index; CNY, Chinese yuan.

**Table 2:** Comparison of baseline comorbidities characteristics between the intervention and control groups.

Variables	Overall (*n* = 57)	Intervention (*n* = 28)	Control (*n* = 29)	χ^2^/Z	*p*
Presence of comorbidities ^a^					
No	29 (50.9)	12 (42.9)	17 (58.6)	1.416	0.234
Yes	28 (49.1)	16 (57.1)	12 (41.4)		
Cancer stage ^a^					
I	5 (8.8)	3 (10.7)	2 (6.9)	0.522	0.771
II	21 (36.8)	11 (39.3)	10 (34.5)		
III	31 (54.4)	14 (50.0)	17 (58.6)		
Number of chemotherapy cycles completed ^c^					
0	28 (49.1)	14 (50.0)	14 (48.3)	1.151	0.819
1	18 (31.6)	8 (28.6)	10 (34.5)		
2	3 (5.3)	1 (3.6)	2 (6.9)		
3	8 (14.0)	5 (17.8)	3 (10.3)		
Type of surgery ^b^					
Laparoscopic surgery	51 (89.5)	24 (85.7)	27 (93.1)	0.228	0.633
Laparotomy	6 (10.5)	4 (14.3)	2 (6.9)		
Chemotherapy regimen ^c^					
FOLFOX	24 (42.1)	12 (42.8)	12 (41.4)	1.392	1.000
FOLFIRI	3 (5.3)	1 (3.6)	2 (6.9)		
Capecitabine	1 (1.8)	0 (0.0)	1 (3.4)		
Others	29 (50.9)	15 (53.6)	14 (48.3)		
Smoking status ^a^					
Never	28 (49.1)	16 (57.1)	12 (41.4)	1.416	0.234
Former/Current	29 (50.9)	12 (42.9)	17 (58.6)		
Drinking status ^a^					
Never	34 (59.6)	19 (67.9)	15 (51.7)	1.540	0.215
Former/Current	23 (40.4)	9 (32.1)	14 (48.3)		
Physical activity level ^a^					
Low	20 (35.1)	12 (42.9)	8 (27.6)	1.459	0.227
Moderate	37 (64.9)	16 (57.1)	21 (72.4)		
High	0 (0.0)	0 (0.0)	0 (0.0)		

Data are shown as *n* (%) or mean ± SD. ^a^ Chi-squared test. ^b^ Chi-squared test with continuity correction. ^c^ Fisher’s exact test. FOLFOX, a chemotherapy regimen consisting of fluorouracil, leucovorin, and oxaliplatin, along with other oxaliplatin-based treatment protocols. FOLFIRI, a chemotherapy regimen consisting of fluorouracil, leucovorin, and irinotecan, along with other irinotecan-based treatment protocols.

**Table 3:** Comparison of baseline lifestyle characteristics between the intervention and control groups.

Variables	Overall (*n* = 57)	Intervention (*n* = 28)	Control (*n* = 29)	χ^2^	*p*
Smoking status					
Never	28 (49.1)	16 (57.1)	12 (41.4)	1.416	0.234
Former/Current	29 (50.9)	12 (42.9)	17 (58.6)		
Drinking status					
Never	34 (59.6)	19 (67.9)	15 (51.7)	1.540	0.215
Former/Current	23 (40.4)	9 (32.1)	14 (48.3)		
Physical activity level					
Low	20 (35.1)	12 (42.9)	8 (27.6)	1.459	0.227
Moderate	37 (64.9)	16 (57.1)	21 (72.4)		
High	0 (0.0)	0 (0.0)	0 (0.0)		

Data are shown as *n* (%) or mean ± SD. Chi-squared test.

**Table 4:** Comparison of plasma cytokines between the intervention and control groups.

Variable	Intervention		Control		t/Z	*p*
IL-6 (pg/mL)						
T1	7.35 ± 0.63		7.16 ± 0.98		0.885	0.380 ^a^
T2	6.96 ± 0.88		7.28 ± 1.08		–1.242	0.220 ^a^
∆IL-6	–0.40 ± 0.65		0.12 ± 0.40		–3.634	0.001 ^a^
*t*	3.258		1.639			
*p*	0.003 ^c^		0.113 ^c^			
IL-10 (pg/mL)						
T1	17.59 ± 0.87		17.64 ± 0.82		–0.239	0.812 ^a^
T2	17.90 ± 0.93		17.49 ± 0.81		1.767	0.083 ^a^
∆IL-10	0.31 ± 0.68		–0.15 ± 0.46		2.996	0.004 ^b^
*t*	–2.456		–1.716			
*p*	0.021 ^c^		0.098 ^c^			

Data are shown as mean ± SD. ^a^ Independent samples *t*-test. ^b^ Mann−Whitney U test. ^c^ Paired *t*-test. IL-6, interleukin 6; IL-10, interleukin 10. The change (∆) was defined as the post-intervention value minus the baseline value for the same individual.

**Table 5:** Comparison of BMI and MNA scores between the intervention and control groups.

Variable	Intervention	Control	t/Z	*p*
BMI				
T1	21.67 ± 2.72	21.08 ± 2.99	0.788	0.434 ^a^
T2	22.40 ± 2.79	20.68 ± 2.19	2.608	0.012 ^a^
∆BMI	0.32 (0.00, 1.65)	0.00 (–0.75, 0.66)	–2.143	0.032 ^b^
Z	–1.272	0.635		
*p*	0.203 ^d^	0.525 ^d^		
MNA				
T1	22.33 ± 2.31	21.85 ± 3.00	0.681	0.383 ^b^
T2	23.17 ± 2.36	21.79 ± 2.21	2.276	0.027 ^a^
∆MNA	0.84 ± 1.60	–0.05 ± 1.55	2.141	0.037 ^a^
Z/t	–2.562	0.180		
*p*	0.010 ^d^	0.859 ^c^		

Data are shown as mean ± SD or median (25th, 75th percentile). ^a^ Independent samples *t*-test. ^b^ Mann−Whitney U test. ^c^ Paired *t*-test. ^d^ Wilcoxon test. BMI, Body Mass Index; MNA, Mini Nutritional Assessment; The change (∆) was defined as the post-intervention value minus the baseline value for the same individual.

**Table 6:** Comparison of FACT-C scores between the intervention and control groups.

	Intervention	Control	t/Z	*p*
T1	92.91 ± 14.75	84.92 ± 19.07	1.765	0.083 ^a^
T2	97.52 ± 15.76	86.47 ± 16.91	2.551	0.014 ^a^
∆FACT-C scores	4.61 ± 6.34	1.55 ± 5.57	1.940	0.058 ^a^
*t*	–3.849	−1.500		
*p*	<0.001 ^b^	0.145 ^b^		

Data are shown as mean ± SD. ^a^ Independent samples *t*-test. ^b^ Paired *t*-test. FACT-C, The Functional Assessment of Cancer Therapy-Colorectal; The change (∆) was defined as the post-intervention value minus the baseline value for the same individual.

## Data Availability

Due to privacy restrictions, the data are available from the corresponding author upon request.

## Supplementary Materials

The following Supplementary Materials are available for download at: https://www.mdpi.com/article/10.3390/nu17132203/s1, Table S1. Criteria for the definition of frailty developed by Fried et al. [30]; Table S2. Comparison of frailty scores between the intervention and control groups. Table S3. Daily Dietary Intake Reference for Chinese Colorectal Cancer Patients Undergoing Chemotherapy: Informed by the Dietary Inflammatory Index (DII), the Chinese Food Pagoda 2022, and Colorectal Cancer Nutrition Guidelines.

## Abbreviations

The following abbreviations are used in this manuscript:

CRC	Colorectal cancer
DII	Dietary inflammatory index
FP	Fried frailty phenotype
BMI	Body mass index
QoL	Quality of life
MNA	Mini nutritional assessment
IL-6	Interleukin-6
IL-10	Interleukin-10
RCT	Randomized controlled trial
IPAQ-SF	International Physical Activity Questionnaire Short Form
MUFA	Monounsaturated fatty acids
PUFA	Polyunsaturated fatty acids
FACT-C	Functional Assessment of Cancer Therapy-Colorectal
SD	Standard deviation
CNY	Chinese yuan
FOLFOX	Chemotherapy regimen consisting of fluorouracil, leucovorin, and oxaliplatin, along with other oxaliplatin-based treatment protocols
FOLFIRI	Chemotherapy regimen consisting of fluorouracil, leucovorin, and irinotecan, along with other irinotecan-based treatment protocols
