# Supplementary material for: Effects of 12-Week Dietary Inflammatory Index-Based Dietary Education on Frailty Status in Frail Patients with Colorectal Cancer: A Randomized Controlled Trial
Source: Nutrients. 2025 Jul 1;17(13):2203. doi: 10.3390/nu17132203 (PMC12252281; doi:10.3390/nu17132203)
Supplement: Supplementary file 1 [file nutrients-17-02203-s001.zip › nutrients-3713292-supplementary.pdf]

Table S1 Criteria for the definition of frailty developed by Fried *et al*.

**The Fried Frailty Criteria**	**Detailed Description**			
Unintentional weight loss	Unintentional loss of 4.5kg (10Ibs) in the year before the current evaluation or unintentional weight loss of at least 5% of the previous year's body weight			
Self-reporting exhaustion	Evaluation of two statements of the CES-D scale<br>(a) I felt that everything I did was an effort<br>(b) I could not get going<br>Criterion positive if at least one condition is present for 3 days or more during the last week.			
Weakness (grip strength)	Grip strength of the dominant hand, using hand-held dynamometer			
	BMI/male	Cutoff (kg)	BMI/female	Cutoff (kg)
	≤ 24	≤ 29	≤ 23	≤ 17
	24.1-26	≤ 30	23.1-26	≤ 17.3
	26.1-28	≤ 30	26.1-29	≤ 18
	> 28	≤ 32	> 29	≤ 21
Slowness (walking speed)	Cutoff for time to walk 4.57 m at usual pace (static protocol)			
	Height/male (cm)	Cutoff (s)	Height/female (cm)	Cutoff (s)
	≤ 173	≥ 7	≤ 159	≥ 7
	> 173	≥ 6	> 159	≥ 6
Low physical activity level	Assessed by weekly energy expenditure by using the IPAQ-SF: 1 week's activity < 600 MET-min/week			

CES-D, Center for Epidemiological Studies Depression; IPAQ-SF, International Physical Activity Questionnaire-Short Form

Table S2 Comparison of frailty scores between the intervention and control groups.

	Intervention	Control	*Z*	*p*
FP scores				
T1	4.00 (3.00, 4.00)	4.00 (3.00, 4.00)	–0.915	0.360 [a]
T2	3.00 (3.00, 4.00)	4.00 (3.00, 5.00)	–1.904	0.057 [a]
ΔFP	–1.00 (–2.00, 1.00)	0.00 (–1.50, 1.00)	–1.069	0.285 [a]
Z	–1.681	–0.439		
p	0.093 [b]	0.661 [b]		

[a] Mann–Whitney U test. [b] Wilcoxon test. FP, The Fried Frailty Phenotype.

Table S3. Daily Dietary Intake Reference for Chinese Colorectal Cancer Patients Undergoing Chemotherapy: Informed by the Dietary Inflammatory Index (DII), the Chinese Food Pagoda 2022, and Colorectal Cancer Nutrition Guidelines

Food Category	Recommended Intake	Colorectal Cancer Dietary Considerations
Water	1500–1700 mL/day	Ensure adequate hydration.
Grains	200–300 g/day	Encourage intake; use refined grains if gastrointestinal tolerance is reduced during treatment.
Whole grains and legumes	50–150 g/day	Encourage intake; adjust if bloating, gas, or ostomy is present.
Tubers	50–100 g/day	Encourage intake; tolerability varies and it should be introduced gently during gastrointestinal distress.
Vegetables	300–500 g/day	Encourage diverse intake; prioritizing dark-colored ones for more antioxidants and fiber.
Fruits	200–350 g/day	Encourage intake; choose soft, peeled, or cooked fruits if digestion is an issue.
Animal-based	120–200 g/day	Encourage intake; limit red and processed

foods		meats.
Eggs	50 g/day	Encourage intake; well tolerated when fully cooked.
Seafood	40–75 g/day	Encourage intake; avoid raw forms and monitor digestive tolerance.
Poultry	40–75 g/day	Encourage intake; steam or stew for better digestion.
Red meat	<50 g/day	Limit intake; choose lean cuts and small portions if consumed.
Processed meats	Avoid	Avoid intake; may worsen gastrointestinal symptoms and inflammation.
Fried foods	Avoid	Avoid intake; can worsen gastrointestinal symptoms.
Soy and soy products	15–25 g/day	Encourage intake; prefer tofu or soy milk to reduce gas, and introduce gradually.
Nuts and seeds	70 g/week	Encourage intake; nut butters may be better tolerated than whole nuts during treatment.
Milk and dairy products	300–500 g/day	Encourage intake; choose low-fat versions to help maintain protein intake during poor appetite.
Cooking oil	25–30 g/day	Use plant oils in moderation; prefer olive or canola oil.
Salt	<5 g/day	Strictly limit intake; watch for hidden salt in processed foods.
Added sugars	<25 g/day	Minimize intake; low nutritional value and may displace healthier foods.
Alcohol	Avoid	Avoid intake; impairs immunity and causes dehydration.
